# Acquired CCDC6-RET Fusion After First-Line Osimertinib in Epidermal Growth Factor Receptor (EGFR)-Mutant Lung Adenocarcinoma: A Case Report

**DOI:** 10.7759/cureus.101571

**Published:** 2026-01-14

**Authors:** Ana Raquel S Afonso, Luisa Nascimento, Margarida Cruz, Teresa Gomes, Bebiana Conde

**Affiliations:** 1 Department of Pulmonology, Unidade Local de Saúde de Trás-os-Montes e Alto Douro, Vila Real, PRT

**Keywords:** acquired resistance, ccdc6-ret, egfr-mutant nsclc, osimertinib, ret rearrangement, selpercatinib

## Abstract

Osimertinib is the standard first-line treatment for advanced non-small cell lung cancer (NSCLC) with sensitizing epidermal growth factor receptor (EGFR) mutations. Despite initial responses, most patients eventually develop acquired resistance through heterogeneous molecular mechanisms, including activation of bypass signaling pathways. RET gene fusions represent a rare and still underreported cause of acquired resistance, for which clinical evidence supporting combined targeted treatment remains limited.

We report a case of EGFR exon 19-mutant lung adenocarcinoma that developed an acquired CCDC6-RET fusion following treatment with first-line osimertinib, identified through repeat molecular profiling at disease progression. Radiological assessment revealed a heterogeneous pattern of response, with sustained control of the primary lung lesion and improvement in some metastatic sites, alongside progression in others, predominantly in the liver, supporting the hypothesis of intratumoral heterogeneity. A combined strategy with continued osimertinib and addition of the selective RET inhibitor selpercatinib was pursued based on biological rationale; however, the short duration of combined treatment precluded a meaningful assessment of clinical benefit.

This case highlights the importance of molecular reassessment at progression to identify rare but actionable resistance mechanisms that may significantly influence therapeutic strategy in EGFR-mutant NSCLC.

## Introduction

Osimertinib has significantly improved outcomes as the standard first-line therapy for patients with advanced non-small cell lung cancer (NSCLC) harboring sensitizing epidermal growth factor receptor (EGFR) mutations [1]. In real-world clinical practice, first-line osimertinib has been associated with a median progression-free survival of approximately 20 months in patients with EGFR-mutant advanced NSCLC [2]. Nevertheless, disease progression eventually occurs, and acquired resistance inevitably develops through diverse mechanisms, including a broad range of heterogeneous molecular alterations [3-5]. While MET amplification represents the most frequently reported bypass pathway, rarer alterations such as RET fusions are increasingly recognized as clinically actionable events mediating resistance to EGFR tyrosine kinase inhibitors (TKIs), reported in approximately 1%-2% of cases and representing potentially targetable alterations [6-8]. However, evidence supporting combined EGFR and RET inhibition as a therapeutic strategy in this setting remains limited, particularly in real-world clinical practice [9-12].

## Case presentation

**Figure 1:**
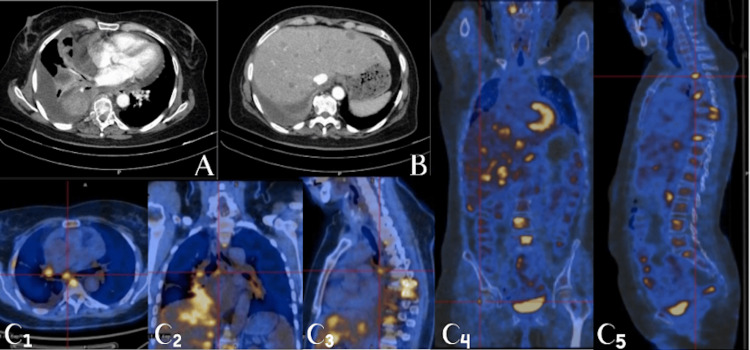
Baseline imaging at diagnosis (A) Contrast-enhanced chest CT demonstrating the primary lung adenocarcinoma in the right lung, associated with bronchial wall thickening and narrowing of the right lower lobe bronchus, resulting in obstructive atelectasis of the right middle and lower lobes. (B) Contrast-enhanced abdominal CT showing multiple hypodense hepatic lesions consistent with hepatic metastatic disease. (C1-C5) Whole-body FDG PET-CT images, including axial, coronal, and sagittal maximum intensity projection views, demonstrating hypermetabolic activity of the primary lung lesion and widespread metabolically active metastatic disease, including extensive osseous involvement, as well as mild FDG uptake along focal pleural thickening adjacent to a small-volume pleural effusion, consistent with Stage IVB disease.

We report the case of a 61-year-old never-smoking woman diagnosed with Stage IVB lung adenocarcinoma, with metastatic disease involving the pleura, liver, and bone (Figure 1). At diagnosis, comprehensive molecular profiling was performed on bronchoscopic biopsy specimens, including a DNA-based next-generation sequencing (NGS) panel for actionable mutations and a targeted RNA-based fusion panel (QIAseq RNAscan Human Lung Cancer Panel). The DNA-based analysis identified an EGFR exon 19 deletion-insertion mutation (EGFR c.2237_2255delinsT; p.E746_S752delinsV) and a concomitant TP53 exon 5 missense mutation (c.527G>T; p.(C176F)). RNA-based fusion analysis did not detect any gene rearrangements, including RET fusions. PD-L1 expression was intermediate (10%-25%). First-line treatment with osimertinib at a dose of 80 mg once daily was initiated in July 2024 and resulted in rapid clinical improvement and partial radiologic response according to RECIST version 1.1 criteria, confirming EGFR dependency. In addition to systemic therapy, the patient received antalgic palliative radiotherapy for spinal metastases and subsequently underwent posterior spine fixation due to neurologic symptoms.

After eight months of treatment, the patient developed multifocal disease progression, predominantly involving the liver. At progression, a liver biopsy confirmed metastatic adenocarcinoma compatible with pulmonary origin, supported by histopathological examination and immunohistochemical positivity for CK7, CK20, Napsin-A, and TTF-1. Comprehensive DNA- and RNA-based NGS demonstrated persistence of the original EGFR exon 19 mutation and revealed a de novo CCDC6-RET fusion (CCDC6 exon 1-RET exon 12), classified as a rare, pathogenic, and targetable resistance mechanism consistent with an acquired mechanism of resistance.

**Figure 2:**
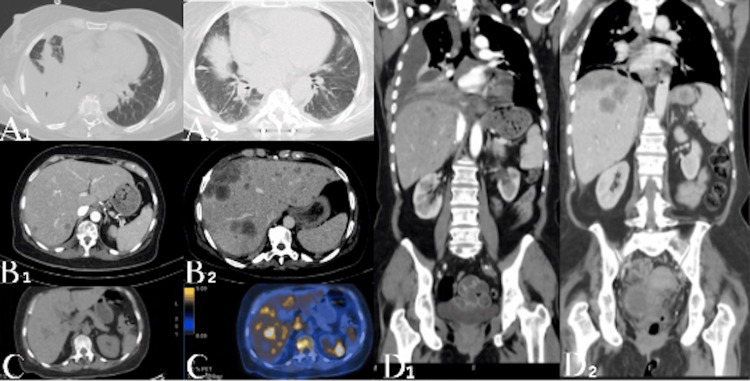
Radiologic assessment demonstrating a heterogeneous response to first-line osimertinib (A1-A2) Contrast-enhanced chest CT images at baseline (A1) and after treatment (A2) demonstrating regression of the primary lung lesion, with improvement of obstructive atelectasis involving the right middle and lower lobes. (B1-B2) Contrast-enhanced abdominal CT images at baseline (B1) and after treatment (B2) showing progression of multifocal hepatic metastatic disease. (C) Follow-up whole-body FDG PET-CT demonstrating a hypermetabolic left adrenal lesion, consistent with new metastatic involvement, along with metabolically active hepatic metastases. (D1-D2) Contrast-enhanced thoracoabdominal CT images at baseline (D1) and after treatment (D2) illustrating a heterogeneous response pattern, with concomitant regression of pulmonary disease and progression of hepatic metastases.

Overall, radiologic assessment was consistent with a heterogeneous response to therapy, characterized by sustained response of the primary lung lesion and most bone lesions, alongside multifocal disease progression involving the liver, with the emergence of a new left adrenal metastasis (Figure 2).

Based on the molecular findings and in the absence of alternative targetable resistance mechanisms, a combined targeted approach was pursued, maintaining osimertinib and adding the selective RET inhibitor selpercatinib, as previously reported in case reports and small series of acquired RET fusion-mediated resistance [8,13,14]. This off-label treatment strategy was discussed and agreed upon within a multidisciplinary tumor board. Due to administrative delays related to off-label approval, initiation of selpercatinib was delayed, while osimertinib was continuously maintained.

Despite this molecularly matched therapeutic strategy, the patient experienced rapid clinical deterioration related to progressive disease. After approximately two weeks of selpercatinib, the patient was hospitalized with suspected cholangitis in the context of malignant biliary obstruction due to extensive hepatic disease. The short duration of treatment precluded a meaningful assessment of therapeutic efficacy and did not suggest combination treatment failure.

## Discussion

RET gene rearrangements represent an uncommon mechanism of acquired resistance to EGFR TKIs, occurring in approximately 1%-2% of cases treated with osimertinib, but are still underreported [4,8,12]. The detected fusion is associated with sensitivity to RET-directed therapies, including other multikinase inhibitors such as vandetanib, cabozantinib, and lenvatinib, as well as newer selective RET inhibitors, namely selpercatinib and pralsetinib [12].

Functional studies have demonstrated that CCDC6-RET fusions can confer resistance to EGFR inhibition, while dual EGFR and RET blockade may restore downstream pathway suppression and tumor sensitivity. These alterations constitute a biologically plausible bypass pathway and provide a rationale for combined EGFR and RET inhibition [12,15,16].

In the clinical setting, published case reports and prospective cohorts have shown that combining osimertinib with selective RET inhibitors such as selpercatinib can provide meaningful clinical benefit with an acceptable safety profile [8,13,14,17]. However, evidence remains limited, and real-world barriers may significantly influence outcomes and complicate the assessment of therapeutic efficacy.

Although combined EGFR and RET inhibition has generally shown a manageable safety profile, selpercatinib has been associated with several treatment-related adverse events. The most frequently reported adverse events include edema, diarrhea, fatigue, dry mouth, abdominal pain, constipation, hypertension, rash, nausea, and headache [18]. Grade 3-4 toxicities most commonly consist of hypertension and elevations in alanine aminotransferase and aspartate aminotransferase, which may, in some cases, lead to dose reduction or treatment interruption [19]. Overall, available data support selpercatinib as an effective and generally well-tolerated targeted therapy in RET fusion-positive NSCLC.

In the present case, the clinical course did not allow for a meaningful evaluation of treatment tolerability. The patient experienced rapid clinical deterioration driven by progressive hepatic disease with malignant biliary obstruction. Laboratory abnormalities were predominantly cholestatic, with elevations in gamma-glutamyl transferase and alkaline phosphatase rather than transaminases, supporting disease-related biliary obstruction rather than treatment-related hepatotoxicity as the main contributor to clinical decline. Importantly, initiation of selpercatinib was delayed due to regulatory and reimbursement constraints related to its off-label use, which likely limited the opportunity to assess therapeutic benefit despite appropriate identification of actionable resistance mechanisms.

From a practical perspective, this case emphasizes the importance of close clinical and radiologic monitoring during osimertinib therapy, as disease progression is expected over time. The emergence of progression should prompt a timely molecular reassessment to identify potential resistance mechanisms. While liquid biopsy may play a complementary role, particularly when tissue sampling is not feasible, its sensitivity for detecting gene fusions remains limited, and a negative result does not exclude the presence of actionable resistance alterations. Therefore, whenever clinically feasible, tissue rebiopsy of the most accessible progressing lesion remains the preferred approach. In the present case, progressive hepatic metastases allowed tissue sampling and comprehensive molecular profiling, leading to the identification of an acquired, targetable RET fusion that directly informed therapeutic decision-making.

## Conclusions

This case highlights the importance of rebiopsy and repeat molecular profiling at the time of progression under targeted therapies to identify emerging mechanisms of resistance and guide personalized treatment strategies. The observed dual pattern of response raises the hypothesis of intratumoral heterogeneity, with resistant tumor clones harboring additional genetic alterations. Although rare, acquired RET fusions should be considered within the spectrum of resistance mechanisms to third-generation EGFR TKIs in EGFR-mutant NSCLC and represent clinically relevant, actionable therapeutic targets with the potential to significantly influence treatment strategy. Although combined EGFR and RET inhibition represents a biologically rational approach supported by emerging evidence, real-world factors may limit the ability to fully assess clinical benefit in individual cases. Early recognition of targetable resistance mechanisms, coupled with timely access to matched therapies, remains critical to translating molecular insights into meaningful clinical outcomes.
